# Mitigating methane emissions in grazing beef cattle with a seaweed-based feed additive: Implications for climate-smart agriculture

**DOI:** 10.1073/pnas.2410863121

**Published:** 2024-12-02

**Authors:** Paulo Meo-Filho, John F. Ramirez-Agudelo, Ermias Kebreab

**Affiliations:** ^a^Department of Animal Science, University of California, Davis, CA 95616

**Keywords:** enteric methane, pasture, ruminants, seaweed

## Abstract

The ruminant livestock sector considerably contributes to global greenhouse gas emissions. This study investigates the effectiveness of pelleted bromoform-containing seaweed (*Asparagopsis taxiformis*) (Brominata) as an enteric methane (CH_4_) inhibitor in grazing beef cattle. The primary objective was to assess the impact of this antimethanogenic additive on enteric CH_4_ emissions under real-world farm conditions. Twenty-four beef steers, crossbreeds of Wagyu and Angus, with an average liveweight of 399 ± 21.7 kg, were allocated to two treatment groups: Control and Brominata. These animals underwent regular weigh-ins every 14 d, and measurements of CH_4_, carbon dioxide (CO_2_), and hydrogen (H_2_) emissions were conducted using the GreenFeed system. Statistical analysis was conducted using SAS 9.4, wherein the model incorporated fixed effects for treatment, time, their interaction, and a covariate, while accounting for animal variations as a random effect within each phase. Three phases of bromoform intake were identified: a 3-wk ramp-up phase, a 3-wk optimal phase, and a 2-wk decreasing phase. No differences were observed between the weekly initial and final liveweight, average daily gain, and predicted dry matter intake. During optimal and decreasing phases, average enteric CH_4_ emissions were significantly reduced in steers that received Brominata supplementation compared to those without supplementation (115 vs. 185 g/d, respectively). Additionally, both groups had similar CO_2_ emissions (6.8 vs. 7.2 kg/d), while H_2_ emissions were lower in the control group (3.4 vs. 1.8 g/d). The findings suggest that pelleted bromoform-containing feed additive has the potential to reduce enteric CH_4_ emissions from grazing beef cattle.

## Significance

This study suggests that the addition of pelleted bromoform-containing seaweed (*Asparagopsis taxiformis*) to the diet of grazing beef cattle can potentially reduce enteric methane (CH_4_) emissions (g/d) by an average of 37.7% without adversely impacting animal performance. Considering the substantial contribution of ruminant livestock to global greenhouse gas emissions, particularly CH_4_, a potent short-lived climate pollutant, this research offers a promising avenue for mitigating climate change. The findings may be relevant in the context of growing global demand for livestock products and the urgent need to address the environmental impacts of animal source foods. Thus, this study contributes to the broader efforts aimed at developing more sustainable and environmentally friendly agricultural practices.

Greenhouse gases (GHGs) are primary drivers of climate change, trapping heat in the earth’s atmosphere and causing global temperatures to rise. In recent years, annual emissions have been estimated at approximately 50 billion tons of carbon dioxide (CO_2_) equivalent (1). Livestock supply chains are estimated to account for 14.5% of total human-induced GHG emissions (2), and it is estimated that about 80% of the GHG emissions from livestock and 90% of methane (CH_4_) emissions are derived from ruminant livestock (3). CH_4_ is the second-most prevalent greenhouse gas emitted from human activities contributing to about 16 to 17% of global GHG emissions (1). Unlike CO_2_, which can persist in the atmosphere for centuries, CH_4_ has a much shorter lifespan, typically around 12 y (4). However, during its relatively brief atmospheric presence, CH_4_ is highly effective at trapping heat. CH_4_ exhibits a global warming potential that is approximately 80 times more effective at absorbing heat than CO_2_ over a 20-y period and 27 times over a century (5). Considering that demand for livestock products continue to increase globally and 30% of the world’s anthropogenic CH_4_ emissions arise from enteric sources in ruminant livestock (6), there is a growing focus on mitigating enteric CH_4_ emissions.

CH_4_ production in ruminants occurs as a by-product of the microbial fermentation process in the rumen, where a consortium of bacteria, archaea, protozoa, and fungi break down feedstuffs, primarily carbohydrates (7). Methanogens, a group of archaea, play a critical role in this process by converting hydrogen (H_2_) and CO_2_ into CH_4_, a process that is not only an energy loss for the animal but also detrimental to the environment (8). Comprehensive analyses have been conducted on the biochemistry of rumen fermentation and CH_4_ production in ruminants (9), as well as the microbiological aspects (10, 11). Similarly, a tremendous progress has been made in the development of mitigation practices in the last two decades, which has been comprehensively reviewed (12, 13).

Mitigation strategies can be broadly classified into three main categories: animal efficiency enhancers, rumen modifiers, and methanogenesis inhibitors. In the first category, the focus is on reducing emissions on a product basis, meaning a decrease in emission intensity (grams of CH_4_ per kilogram of product) or yield [grams of CH_4_ per kilogram of dry matter intake (DMI)]. For example, intensification of animal production through enhanced feeding, management, health, and breeding practices increases individual animal productivity and reduces CH_4_ emission intensity per product unit (14). Another tool in this category is genetic selection of low-CH_4_ producing animals, which is expected to reduce CH_4_ yield. Research conducted in New Zealand revealed that a decade-long selective breeding effort focusing on ewes with varying CH_4_ yields was effective, resulting in a 12% variance between the different genetic lines (15). Strategies in the efficiency category are well suited to be deployed in extensive production systems where animals are not normally supplemented, particularly in tropical regions.

In the rumen modifiers category, dietary lipids are a promising strategy for reducing CH_4_ emissions by inhibiting methanogens and protozoa, altering fermentation, and replacing fermentable carbohydrates (16, 17). Their effectiveness depends on the type, source, amount, and fatty acid composition of the supplement (17, 18). Plant secondary compounds like tannins also reduce CH_4_ with 3.65% CH_4_ yield for every 10 g/kg dry matter addition (19) but this also reduces organic matter digestibility by 2.6% requiring a balance between emissions reduction and digestibility. Alternative electron acceptors such as nitrate can reduce CH_4_ production (20) but with risks of toxicity (21).

Among the most effective feed additives are methanogenesis inhibitors like 3-Nitrooxypropanol (3-NOP) (22), and haloform-containing compounds. In vivo experiments in sheep (23), beef (24, 25), and dairy cattle (26, 27) demonstrated that bromoform-containing macroalgae is an effective methanogenesis inhibitor. Iodoform as an antimethanogenic feed additive for dairy cows was also a potent mitigator of CH_4_ emissions, with reductions of up to 66% (28). However, increased doses of iodoform and bromoform led to decreases in DMI and milk production (26, 28).

Various techniques and methodologies are used to measure enteric CH_4_ emissions from ruminants, including gas exchange measurements, air spot sampling, tracer gas, and micrometeorological technologies (29). In this study, we used GreenFeed Emission Monitoring system (C-Lock Inc., South Dakota). While GreenFeed devices are widely recognized for their ability to provide continuous, noninvasive monitoring of enteric CH_4_ emissions, it is important to consider the accuracy and limitations of these measurements, particularly concerning absolute CH_4_ output. The GreenFeed system is effective in detecting differences in CH_4_ emissions between treatment groups, providing robust relative measurements (30). However, the accuracy of absolute CH_4_ values reported by GreenFeed devices can be influenced by factors such as measurement frequency and timing, animal behavior, and environmental conditions (31). Consequently, while relative differences in CH_4_ emissions between groups are reliable, the absolute quantum of these differences may be less accurate. Given this potential variability, conclusions drawn from studies on antimethanogenic feed additives should be interpreted primarily in a comparative context. The observed relative reductions in CH_4_ emissions in the treatment group are meaningful and demonstrate the efficacy of the intervention. However, the exact magnitude of these reductions should be interpreted with caution (30).

The studies investigating antimethanogenic additives have mostly been conducted under controlled conditions with daily supplementation. There is urgent need to develop novel mitigation strategies, especially for pasture-based systems as less than half of identified strategies were relevant for grazing systems (18). Pastoral agriculture, which includes extensive grazing systems, is important for the livelihoods of millions globally, often in regions vulnerable to the impacts of climate change. These systems are not only economically essential but are also deeply intertwined with local cultures and biodiversity conservation. The study’s focus on grazing beef cattle treated with Brominata underlines a critical intersection: the potential to enhance pastoral agriculture’s sustainability and its capacity to contribute to global climate mitigation efforts. Therefore, the objective of this study was to assess the effectiveness of Brominata, a feed additive containing the seaweed *Asparagopsis taxiformis*, in beef cattle grazing on pasture under real-world farm conditions.

## Results and Discussion

### Enteric CH_4_ Production, Yield, and Intensity.

**Fig. 1.**
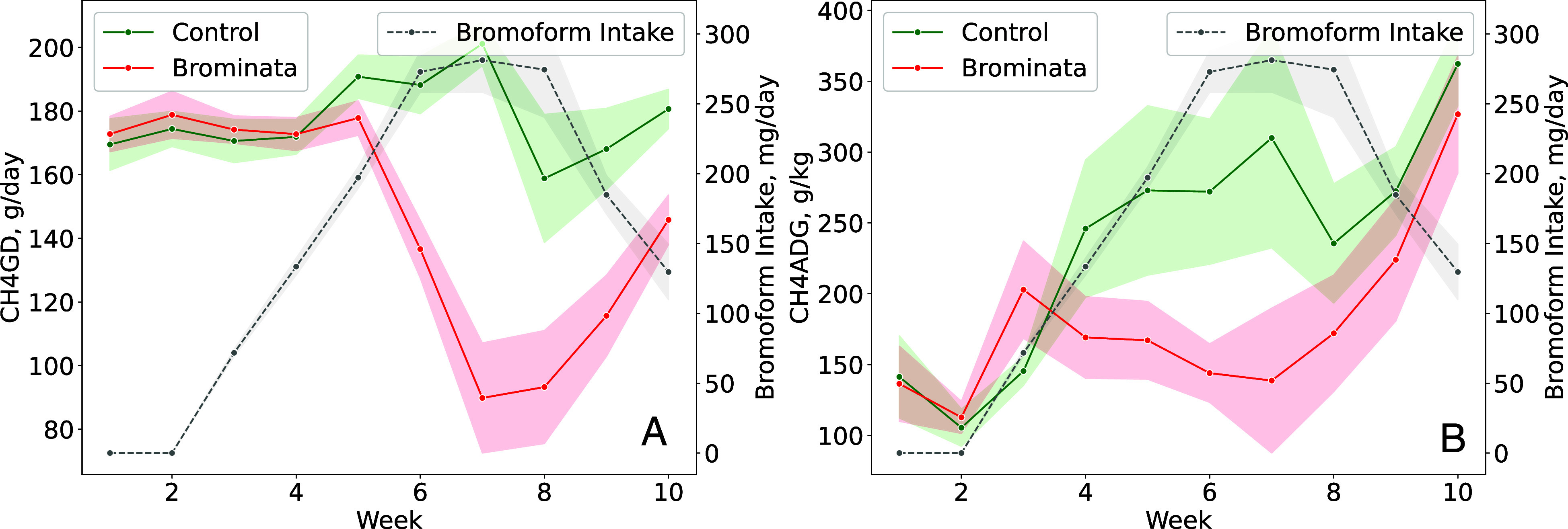
Enteric CH_4_ emissions and bromoform intake of grazing beef steers treated with *A. taxiformis*. (*A*) CH_4_ emissions (CH_4_GD, g/d) and (*B*) CH_4_ emissions per average daily gain (CH_4_ADG, g/kg). Control = 0 mg/kg bromoform, and bromoform treatment = average 193 mg/d. CH_4_ emissions were measured using the GreenFeed system (C-Lock, Inc.). Data are presented as treatment means with SEM; n = 9 (number of independent data points for each mean value).

The changes in enteric CH_4_ emissions (g/d) between the control and treatment groups, alongside bromoform intake (mg/d), are illustrated in Fig. 1*A*. The control group maintained relatively stable CH_4_ emissions, fluctuating between 160 to 200 g/d. In contrast, the treatment group exhibited a notable reduction in CH_4_ emissions within 16 d of introducing the pelleted Brominata. This reduction persisted throughout the subsequent 5-wk period (optimal and decreasing phases), consistently remaining below the control group’s emissions. Additionally, the treatment group’s bromoform intake peaked between weeks 6 and 8 before decreasing slightly, although it still maintained relatively high levels. The decrease in bromoform intake can be largely attributed to the palatability of this compound.

Palatability issues related to halogenated compounds have been well documented in previous studies (26). In our study, we tested various formulations of pelletized Brominata before commencing the experiment, and the formulation used was the most acceptable to the steers. However, there is room for improvement in developing a more palatable formulation of pelleted Brominata to avoid a reduction in feed additive intake. One option could be increasing the level of bromoform concentration in the product so that relatively small amounts of macroalgae need to be supplemented with the diet. This option will also reduce accumulation of inorganic compounds such as iodine and bromine, which may be found in relatively greater concentrations in macroalgae, particularly those harvested from the ocean. In addition, there were small but notable differences in the composition of the pellets for the control and Brominata groups due to processing limitations, which required the use of molasses in the control pellets and distillers dried grains in the Brominata pellets (Table 1). While the quantities of molasses and distillers dried grains used were relatively small, these ingredients are different substrates with distinct nonfiber carbohydrate structures. This difference could potentially influence CH_4_ emissions, even if the impact may be minor.

**Table 1.** Ingredients and chemical composition of the diet fed in the experiment including control and treatment pellets

Ingredient (%)	Brominata pellet	Control pellet	Forage
Wheat mids	64.8	65	–
Distillery solubles	15.0	–	–
Molasses	–	15	–
Bentonite	–	20	–
Brominata	20.0	–	–
Palatability enhancer	0.25	–	–
%DM^*^	Nutritional composition		
DM	86.8	88.7	30.9
CP	15.8	16.5	10.2
TDN	58.3	63.2	58.2
ME MJ/kg	8.95	9.91	8.95
NDF	36.3	40.3	51.8
ADF	21.3	12.6	36.4
Lignin	3.9	4.4	5.0
Crude fat	3.1	3.0	3.9
NFC	–	39.7	23.3
Ash	17.7	14.3	12.7
Ca	0.3	0.3	0.9
P	0.7	0.8	0.3
Mg	0.5	0.4	0.3
K	1.2	1.2	2.4
Na	0.6	0.5	0.04
g/kg	0.0138		
Fe	1.68	1.10	0.174
Mn	0.126	0.123	0.142
Zn	0.078	0.073	0.0147
Cu	0.0138	0.0125	0.0057

^*^DM = dry matter; CP = crude protein; TDN = total digestible nutrients; ME = metabolizable energy; NDF = neutral detergent fiber; ADF = acid detergent fiber; NFC = nonfiber carbohydrates; Ca = calcium; P = phosphorus; Mg = magnesium; K = potassium; Na = sodium; Fe = iron; Mn = manganese; Zn = zinc; Cu = copper.

During the optimal and decreasing phases, pelleted Brominata significantly reduced (*P* ≤ 0.05) average CH_4_ production (g/d) by 37.7%, CH_4_ per live weight (LW) (g/kg LW) by 37.2%, CH_4_ intensity [g/kg, average daily gain (ADG)] by 34.5%, and CH_4_ yield (g/kg predicted DMI, pDMI) by 38.1% compared to the control group. The interaction between treatment and time during bromoform intake (Weeks 3 to 10) was significant (*P* ≤ 0.05) for most variables, except for CH_4_ intensity, highlighting specific weeks (Weeks 6 to 10) where treatment effects were most pronounced. The significance of the covariates across all variables (*P* ≤ 0.05) emphasizes their importance in analyzing CH_4_ emissions (Table 2).

**Table 2.** Effect of pelleted *A. taxiformis* based feed additive (Brominata) on CH_4_, CO_2_, and H_2_ emissions in grazing Wagyu × Angus steers, Means ± SEM

Week^*^	Control	Brominata	Treat.	Time	Covariate	Treat.*Time
CH_4_ (g/d)						
3 to 10	182 ± 3.0	138 ± 5.5	<0.001	<0.001	0.002	<0.001
6	188 ± 9.0	137 ± 9.1	<0.001	–	–	–
7	201 ± 6.9	89.8 ± 17.3	<0.001	–	–	–
8	175 ± 13.7	93.3 ± 17.8	<0.001	–	–	–
9	177 ± 10.5	116 ± 12.8	0.002	–	–	–
10	181 ± 6.2	143 ± 8.3	<0.001	–	–	–
CH_4_ (g/kg LW)						
3 to 10	0.43 ± 0.007	0.32 ± 0.013	<0.001	<0.001	0.041	<0.001
6	0.44 ± 0.019	0.32 ± 0.020	<0.001	–	–	–
7	0.47 ± 0.017	0.21 ± 0.040	<0.001	–	–	–
8	0.41 ± 0.031	0.22 ± 0.041	<0.001	–	–	–
9	0.41 ± 0.023	0.26 ± 0.028	0.002	–	–	–
10	0.41 ± 0.012	0.32 ± 0.017	<0.001	–	–	–
CH_4_ (g/kg ADG)						
3 to 10	304 ± 21.5	207 ± 17.6	0.047	0.003	0.007	0.080
6	298 ± 60.1	150 ± 29.0	0.114	–	–	–
7	380 ± 96.2	149 ± 71.3	0.195	–	–	–
8	293 ± 41.5	172 ± 41.1	0.159	–	–	–
9	294 ± 45.4	224 ± 43.2	0.222	–	–	–
10	356 ± 37.7	333 ± 46.5	0.724	–	–	–
CH_4_ (g/kg pDMI)						
3 to 10	21.3 ± 0.3	16.1 ± 0.7	<0.001	<0.001	0.018	<0.001
6	22.0 ± 1.0	16.0 ± 1.0	<0.001	–	–	–
7	23.5 ± 0.8	10.5 ± 2.0	<0.001	–	–	–
8	20.3 ± 1.5	10.8 ± 2.1	<0.001	–	–	–
9	20.5 ± 1.2	13.3 ± 1.4	0.002	–	–	–
10	20.8 ± 0.6	16.3 ± 0.9	<0.001	–	–	–
CO_2_ (g/d)						
3 to 10	6,967 ± 61.2	6,705 ± 58.1	<0.001	<0.001	<0.001	0.008
6	6,876 ± 119.7	6,877 ± 159.0	0.999	–	–	–
7	7,294 ± 69.1	6,880 ± 138.0	0.025	–	–	–
8	7,549 ± 137.8	7,007 ± 118.9	<0.001	–	–	–
9	7,253 ± 146.4	6,567 ± 144.2	<0.001	–	–	–
10	6,850 ± 155.4	6,391 ± 184.4	0.016	–	–	–
CO_2_ (g/kg pDMI)						
3 to 10	816 ± 6.1	783 ± 6.3	<0.001	<0.001	<0.001	0.004
6	806 ± 13.4	807 ± 19.4	0.972	–	–	–
7	853 ± 12.8	802 ± 13.1	0.016	–	–	–
8	878 ± 13.3	812 ± 13.0	<0.001	–	–	–
9	839 ± 10.0	757 ± 15.8	<0.001	–	–	–
10	789 ± 12.3	730 ± 18.8	0.001	–	–	–
H_2_ (g/d)						
3 to 10	1.4 ± 0.1	2.6 ± 0.2	<0.001	<0.001	0.619	<0.001
6	0.9 ± 0.1	3.4 ± 0.3	<0.001	–	–	–
7	0.9 ± 0.2	4.2 ± 0.5	<0.001	–	–	–
8	3.0 ± 0.7	4.3 ± 0.6	0.151	–	–	–
9	2.5 ± 0.4	3.4 ± 0.5	0.159	–	–	–
10	1.6 ± 0.2	2.1 ± 0.2	0.048	–	–	–
H_2_ (g/kg pDMI)						
3 to 10	0.17 ± 0.02	0.30 ± 0.02	<0.001	<0.001	0.509	<0.001
6	0.11 ± 0.01	0.40 ± 0.04	<0.001	–	–	–
7	0.11 ± 0.02	0.48 ± 0.07	<0.001	–	–	–
8	0.35 ± 0.08	0.50 ± 0.08	0.158	–	–	–
9	0.29 ± 0.05	0.40 ± 0.06	0.165	–	–	–
10	0.18 ± 0.02	0.24 ± 0.02	0.064	–	–	–

^*^Results are presented for the entire experimental period (Weeks 3 to 10) and separately for weeks 6 to 10 (optimal and decreasing bromoform intake phases).

LW = liveweight; ADG = average daily gain; pDMI = predicted DMI.

**Fig. 2.**
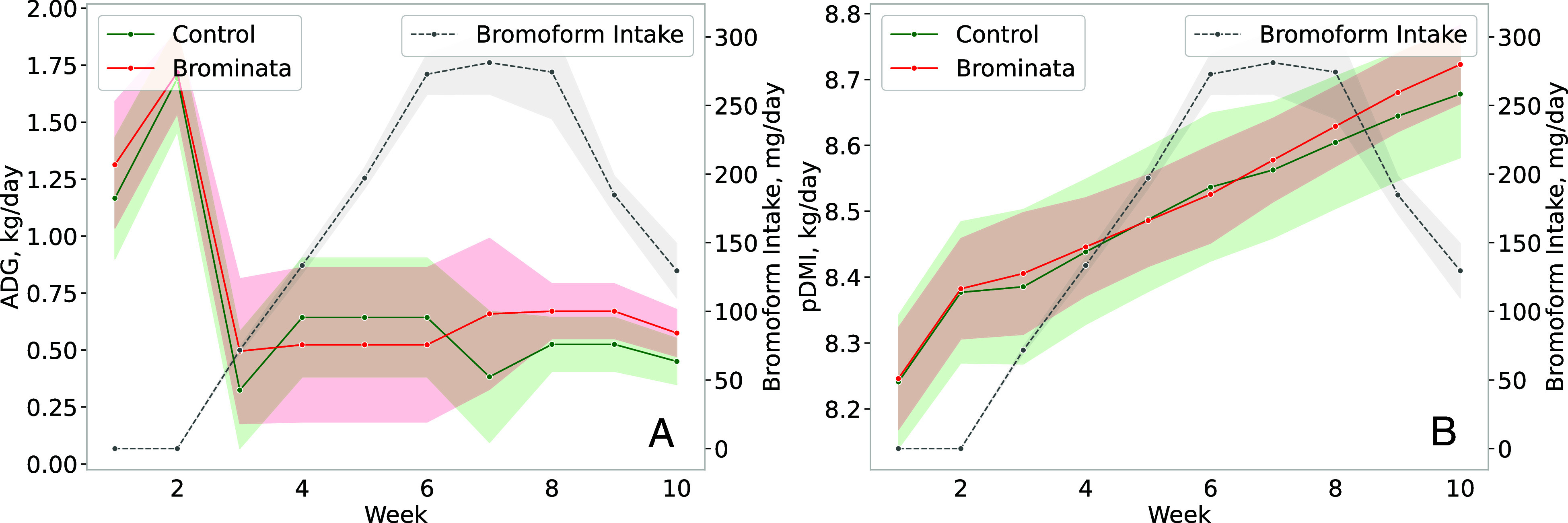
ADG (*A*, kg/d), pDMI (*B*, kg/d) and bromoform intake, of grazing beef steers. Control = 0 mg/kg bromoform, and bromoform treatment = average 193 mg/d. Data are presented as treatment means with SEM; n = 9 (number of independent data points for each mean value).

Encouragingly, we observed no reduction in pDMI (Fig. 2*B*), in contrast to previous studies where a significant drop in intake was reported (26). However, previous reports of DMI suppression often involved relatively higher concentrations of bromoform in the diet. There is evidence suggesting that the reduction in DMI is directly related to bromoform concentration. For example, CH_4_ yield reductions of 80% were reported in lactating dairy cows fed 5.0 g macroalgae/kg DM, but the persistence of this reduction declined in tandem with decreasing bromoform levels in the diet (27). Furthermore, reductions in milk yield and energy-corrected milk were attributed to reduced DMI in previous studies (27). Similar results in lactating dairy cows fed 18.4 g/kg DM *Asparagopsis armata* were reported with a 67% decrease in CH_4_ intensity along with reductions of 38% in DMI and 12% in milk production (26).

The majority of CH_4_ emissions from livestock are attributed to ruminants that graze on pasture in rangeland environments. CH_4_, recognized as a potent short-lived climate pollutant (4), presents an opportunity for achieving short-term benefits in mitigating global warming. This can be achieved by prioritizing reductions in enteric CH_4_ emissions, especially in forage-based systems that may also enable carbon sequestration (32). However, in various regions, the carbon stocks in grasslands would have to surge by as much as 2,000% to counterbalance the warming impact of emissions from existing ruminant systems (33). This suggests that depending solely on carbon sequestration in grasslands as a means to offset these emissions is impractical, which makes emission avoidance from enteric CH_4_ production even more important. Additionally, enteric CH_4_ represents a net energy loss for animals, which could be redirected toward productive purposes, thereby reducing emission intensities (34).

While most research has focused on feedlot systems within the beef sector, there is a pressing need for further development, adaptation, and evaluation of antimethanogenic strategies for grazing systems. Unfortunately, the grazing industry has not been identified as a high-priority market for CH_4_-mitigating products, potentially delaying their adoption in the developing world and global grazing industries (35).

Only a few in vivo studies have explored the use of the macroalgae, *A. taxiformis*, to reduce CH_4_ emissions thus far. These studies examined the impact of varying levels of macroalgae inclusion in total mixed ration feedlot diets, involving Brangus (24) and Angus × Hereford (25) beef steers, Holstein dairy cattle (27), and sheep (23). The results of these studies demonstrated an average CH_4_ reduction of 48%, 63%, 55%, and 50%, respectively. These variable reductions are likely attributable to differences in macroalgae inclusion levels, diet formulations, and variations in bromoform concentrations.

Our study measures enteric CH_4_ emissions from grazing beef cattle fed *A. taxiformis* under real-world farm conditions. In our research, dietary supplementation of grazing beef cattle with bromoform-containing seaweed effectively reduced enteric CH_4_ emissions (Fig. 1). In a related study by Roque et al. (25), the inclusion of 0.25% (~400 mg/d of bromoform) and 0.5% (~680 mg/d of bromoform) of *A. taxiformis* in feedlot diets, along with varying levels of fiber inclusion, resulted in an average reduction of 36.4% and 58.7% in CH_4_ emissions when steers were fed high-forage diet. For comparison, their high-forage diet contained 33.1% neutral detergent fiber, which closely approximates the fiber content of the pasture in our study (36.3%). In our experiment, the average bromoform intake was much lower at 193 mg/d, leading to an overall 37.7% reduction in CH_4_ emissions.

There was a linear relationship between the amount of bromoform consumed and the magnitude of the reduction in enteric CH_4_ production, CH_4_ reduction (%) = 0.21 × bromoform intake (mg/d) – 17.55 (n = 9; R^2^ = 0.65; *P* ≤ 0.05). However, the effect also depended on the phase of the experiment, as similar bromoform intakes had different impacts during the ramp-up and decreasing phases. Based on this relationship, the reduction ranged from about 5% at 100 mg/d intake to about 45% at 300 mg/d intake. For every 100 mg/d increase in bromoform consumption by steers, there was an average reduction of about 20% in CH_4_ emissions. Despite steers in this study consuming half as much bromoform compared to previous trial (25), the magnitude of reduction was similar. However, in another trial when steers in a feedlot consumed about 214 g bromoform/d, enteric CH_4_ reduced by up to 98% (24). The variability in results can be partly attributed to the methodology employed in analyzing bromoform concentration in macroalgae. For instance, when comparing the findings of Kinley et al. (24) and Roque et al. (25), who both utilized the same macroalgae collected in Australia, there is a notable difference in the reported bromoform concentrations: 6.6 mg/g and 7.8 mg/g, respectively. This discrepancy is intriguing because, theoretically, Roque et al.’s (25) measurement should have been lower. The reason is that their study was conducted 3 y after the macroalgae’s harvest and subsequent transportation from Australia to California. Therefore, there is an urgent need to establish standardized procedures for the analysis of bromoform concentration in macroalgae. By doing so, the consistency and reliability in the reported data can be improved, facilitating more accurate comparisons and assessments within the field of research.

Various authors (26, 34), have provided insights into the mode of action of halogenated compounds like bromoform. In brief, these compounds inhibit the methanogenic pathway by suppressing the cobamide-dependent methyl transferase (methyl CoM reductase) at the terminal step of the Archaea synthesis pathway. There is evidence that suggests that halogenated compounds redirect metabolic H_2_ in the rumen, affecting metabolism and microbial communities. Microbial analysis showed a shift in certain bacterial families and a significant impact on major archaeal groups, particularly *Methanobrevibacter* and *Methanosphaera* (36).

Initially, during the ramp-up phase, the introduction of Brominata likely caused a modest reduction in CH_4_ emissions by disrupting methanogenic archaea. This period marked the beginning of microbial community adjustments to bromoform, with methanogens starting to be suppressed. The gradual increase in Brominata intake during this phase may not have reached levels required for optimal CH_4_ suppression. In contrast, during the optimal phase, the consistent and highest intake of Brominata maximized the inhibitory effect on methanogenic archaea, leading to the most pronounced reduction in CH_4_ emissions. However, the decreasing phase showed that, despite a reduction in Brominata intake to levels similar to the ramp-up phase, CH_4_ emissions remained significantly lower than the control group. This sustained reduction could be due to cumulative effects of prolonged bromoform exposure, which may have led to lasting suppression of methanogens. Over time, the rumen microbial ecosystem likely experienced long-term shifts, including a possible reduction in methanogen population or changes in the microbial community structure that continued to suppress CH_4_ production effectively.

### Enteric CO_2_ and H_2_ Emissions.

CO_2_ production and yield decreased (*P* ≤ 0.05) by 4% in the Brominata group compared to the control (Table 2). On the contrary, other studies using *A. taxiformis* or *A*. *armata* (25, 26) did not report differences in CO_2_ production or intensity. However, when adjusted for DMI, Roque et al. (25) observed 13% differences in CO_2_ yield. Studies that used other compounds, such as 3-NOP to inhibit methanogenesis did not report differences in CO_2_ production (34). The small decrease in CO_2_ production and yield in our study may be related to differences in DMI, however, because DMI was predicted, further research with precise measurements of DMI is necessary to confirm this hypothesis. Variations in DMI could influence the metabolic processes in cattle, thereby impacting CO_2_ production. Accurate DMI assessments would provide a clearer understanding of the relationship between feed intake and CO_2_ emissions, ensuring a more comprehensive evaluation of dietary interventions aimed at reducing greenhouse gas emissions from livestock.

**Fig. 3.**
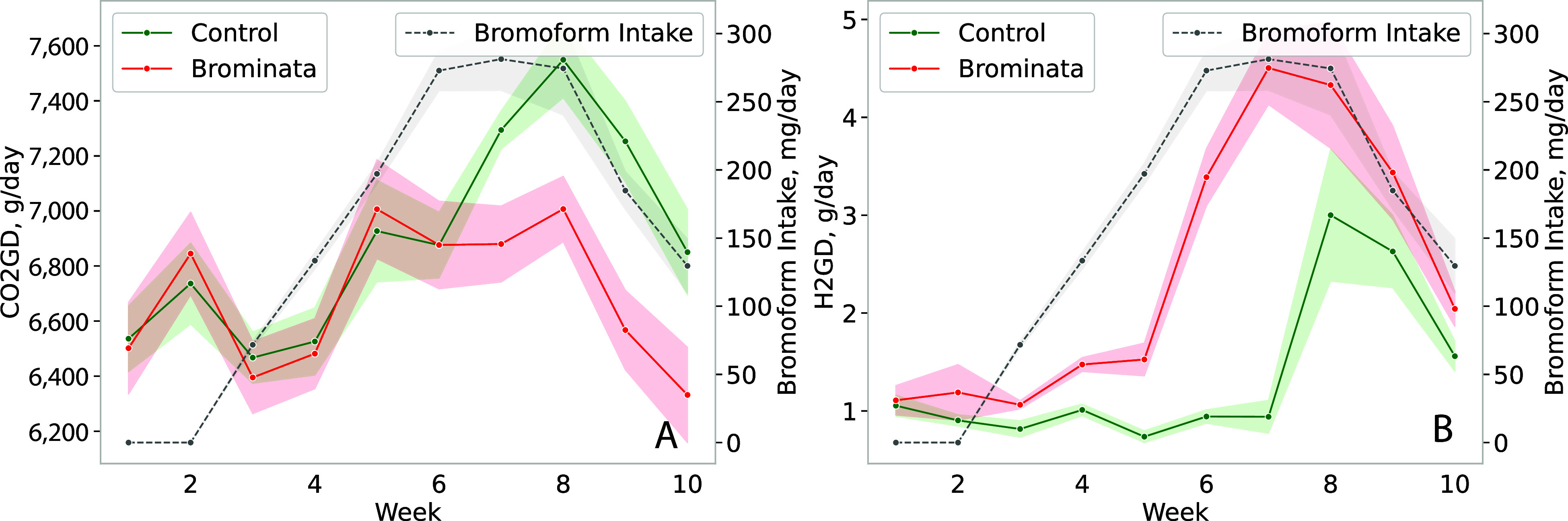
CO_2_ (*A*, CO_2_GD, g/d), H_2_ (*B*, H_2_GD, g/d) emissions and bromoform intake, of grazing beef steers. Control = 0 mg/kg bromoform, and bromoform treatment = average 193 mg/d. CO_2_ and H_2_ emissions were measured using the GreenFeed system (C-Lock, Inc.). Data are presented as treatment means with SEM; n = 9 (number of independent data points for each mean value).

H_2_ emissions were significantly greater (*P* ≤ 0.05) in the Brominata-supplemented steers, with an 85.7% increase in H_2_ production and a 76.5% increase in H_2_ yield. Similar to CH_4_, there was a significant treatment × experimental week interaction for CO_2_ and H_2_ production and yield (Table 2). Regarding CO_2_ emissions, there were no differences in the 6th week, but in the remaining weeks, the control group showed greater CO_2_ production and yield (Fig. 3*A*).There were no differences between treatments for H_2_ production and yield in weeks 8 to 10, except for H_2_ production in week 10, but throughout the experiment (Weeks 3 to 10), the Brominata group emitted significantly more H_2_ than the control group (Fig. 3*B*).

When antimethanogenic feed additives are used, an increase in H_2_ production and yield is often observed, as seen with *Asparagopsis* species in dairy cattle (26) and Brangus feedlot steers (24). This increase in H_2_ yield is also reported with other feed additives that reduce enteric CH_4_ emissions by targeting methanogens (34). An increase in H_2_ emissions is expected when methanogenesis is inhibited, as this pathway normally consumes H_2_ within the rumen (27). It is also suggested that an increase in H_2_ emissions is due to the thermodynamic inhibition of nicotinamide adenine dinucleotide + hydrogen (NADH) oxidation in fermentative microbes in the rumen (37). During the feed fermentation process, H_2_ is generated by fungi, protozoa, and bacteria. While this H_2_ is typically consumed by rumen methanogenic archaea, under significant methanogenesis inhibition, it may be expelled by the ruminant, possibly as an alternative mechanism to eliminate excess H_2_ from the rumen (24). There is a potential within the rumen environment for the utilization of excess H_2_ by anaerobic facultative autotrophs, which are capable of harnessing H_2_ as an electron donor. This utilization of excess H_2_ could lead to concurrent increases in productivity.

Inhibited methanogenesis also leads to an increased partial pressure of H_2_ in the rumen, which in turn causes thermodynamic inhibition of NADH oxidation. The inhibition of NADH oxidation has been simulated to lead to notable shifts in volatile fatty acids (VFA) proportions. Specifically, the proportion of acetate decreases while the proportions of propionate and butyrate increase (37). These shifts in VFA proportions are aligned with in vivo observations from other studies (23, 24, 27). The decrease in acetate and the increase in propionate and butyrate suggest a shift in the ruminal fermentation pathway, potentially contributing to the observed changes in productivity in some studies.

### Emission Intensity Parameters.

There was no statistical difference between treatments for liveweight at the end of the experiment (440 ± 4.4 kg) and for ADG (0.55 ± 0.059 kg/d) throughout the experiment (Weeks 3 to 10). The ADG observed in our experiment falls within a similar range to that of other studies. For instance, in a study conducted by Baker et al. (38), which evaluated F1 crosses of Wagyu and Angus steers and heifers on a continuous grain-based diet or forage feeding to simulate grazing, an ADG of 0.49 kg/d was reported for the forage-fed group.

The absence of significant differences in ADG between the treatment and control groups aligns with findings from a study that investigated sheep fed *A. taxiformis* at a rate of 78.4 g/kg DM (23). This study reported an 80% reduction in CH_4_ yield with no discernible effects on ADG or DMI. In contrast, another study involving beef steers fed 3.7 g/kg DM of *A. taxiformis* reported a 22% increase in ADG (24). Similarly, beef cattle fed *A. taxiformis* at concentrations ranging from 4.9 to 9.8 g/kg DM exhibited 7 to 14% improvements in feed conversion efficiency (25). The lack of a statistically significant improvement in ADG in our experiment may be attributed to the relatively shorter experimental duration and a potential lack of statistical power to detect subtle changes in ADG. However, the Brominata group exhibited higher ADG than the control group from weeks 7 to 10 (Fig. 2*A*).

### Implications and Future Direction.

This study’s key finding—that bromoform-containing seaweed can reduce CH_4_ emissions (g/d) in cattle by an average of 37.7%—is an advancement in reducing the environmental impact of animal source foods, particularly in pasture-based systems. This is especially relevant in the context of the increasing global emphasis on sustainable food systems. The study not only contributes to making animal-sourced foods more ecofriendly but also enhances their environmental competitiveness relative to plant-based alternatives, especially concerning CH_4_ emissions. There are several promising directions for future research. It is crucial to explore the long-term effects and scalability of this dietary intervention, its applicability to other ruminant species, and its economic and social implications. Understanding how the inclusion of seaweed supplements in livestock diets is perceived by farmers, consumers, and markets is essential for its widespread adoption. Additionally, a comprehensive sustainability assessment, encompassing environmental, nutritional, economic, and social dimensions, would provide a more holistic view of the comparative benefits of enhanced animal-sourced foods and plant-based alternatives. Finally, the development of supportive policy and regulatory frameworks is key to facilitating the adoption of these sustainable practices in livestock farming, ensuring food safety and quality while incentivizing farmers to adopt these innovative strategies. This research thus opens up pathways for enhancing the sustainability of animal-sourced foods and may shift some of the current narratives in the debate between animal and plant-based diets.

## Conclusion

In conclusion, our study indicates that bromoform-containing seaweed (*A. taxiformis*) can be effective in reducing enteric CH_4_ emissions from grazing beef cattle. The observed 37.7% average reduction in CH_4_ production, achieved without compromising animal performance, suggests a promising approach for mitigating the environmental impact of livestock farming. These findings contribute to the understanding of dietary modifications as a potential strategy for reducing greenhouse gas emissions in the agricultural sector. This study adds to the existing body of knowledge and suggests areas for further research in sustainable livestock farming.

## Materials and Methods

### Experimental Design, Animals, Treatments, and Emission Measurements.

The study was conducted at the Matador Ranch and Cattle’s Selkirk division in Dillon, Montana, USA, situated in a semiarid climate with cold, dry winters and hot, wet summers. A power analysis using a two-sample means test with a significance level of 0.05 and a desired power of 0.95, based on a CH_4_ yield of 21 (g/kg DMI) with a SD of 3 and a 20% CH_4_ yield difference between treatment and control, recommended 12 animals per group. Therefore, 24 Wagyu × Angus steers, approximately 15 mo old and with an initial average weight of 399 ± 21.7 kg, were sourced from the ranch’s herd. The steers were randomly allocated into two groups: a control group and a group receiving Brominata. The study followed a completely randomized design over 70 d, including a 2-wk covariate period.

The steers in the control group received a pellet, composed of 65% wheat mids, 15% molasses, and 20% bentonite (CHS Nutrition, Great Falls, Montana, USA) while the Brominata group received pellet mixed Brominata (20%), distillery solubles (15%), wheat mids (65%), a palatability enhancer (Inhace, 0.25%, Qualitechtm, Chaska, Minnesota, USA), molasses coating, and wheat mids dusting (Table 1). Pelleted Brominata was purchased from Blue Ocean Barns (Kailua Kona, HI, USA). The bromoform concentration in the pellets was 1.4 mg g/dry matter and was quantified by GCMS analysis according to Romanzzi et al. (39).

CH_4_, CO_2_, and H_2_ gas emissions from steers were measured using the GreenFeed system [C-lock Inc., Rapid City, SD, USA; (31, 40)], which also administered treatments to the animals. Two GreenFeed pasture systems with dual hoppers independently delivered specific treatments to each steer identified by the electronic identification ear tag. To acclimate the steers, an adaptation protocol gradually increased Brominata in the feed mix until the full dose was reached, using various settings and Brominata pellet inclusions.

Both initial liveweight (ILW) and final liveweight (FLW) were measured at the beginning and end of each 2-wk period throughout the experiment. Specifically, FLW was used to assess the biweekly average daily gain (ADG) by comparing FLW to the ILW of the same 2-wk period. To estimate weekly weights from the biweekly data, linear interpolation was used. This method involved calculating the midpoint weight of each 2-wk interval, effectively creating a weekly dataset. DMI was estimated using the prediction model proposed by Anele et al. (41):

pDMI = 0.0167 × FLW + 8.12 × NELm – 3.00 × NELm2 – 3.63, taking into account the weekly FLW and the net maintenance energy (ELm), which was based on the value 0.32 MJ/BW^0.75^ (empty body weight), proposed by the NRC (42) for animals in this category.

The initial 14 d of the study served as a covariate period. Throughout the experiment, the GreenFeed units were accessible to steers, allowing up to three feeding periods per animal each day, spaced at least 6 h apart. In each feeding period, up to eight feed drops of approximately 30 g of pellets were dispensed at 35-s intervals. The average daily intake was 504 g for the Control group and 441 g for the Brominata group. While this difference might raise concerns about its potential impact on the responses, it is important to emphasize that pasture makes up more than 95% of the total feed intake. Given that enteric CH_4_ emissions are positively correlated with the fiber content in the diet, and the diet is predominantly pasture-based, the small difference in pellet intake is unlikely to significantly affect the outcomes. However, it is also important to acknowledge that with the limited sample size, there is a potential risk of a Type II error due to the inherent variability in DMI estimates. This risk should be considered when interpreting the results. A total of 3,494 individual feeding periods and corresponding gas samples were recorded. Weekly standard gas calibrations and monthly CO_2_ recovery calibrations were conducted, with air filters maintained weekly. The gas emissions calculation procedure is detailed in Martin et al. (43).

### Grassland and Forage Sampling.

The study was conducted in an intensive irrigated pasture area where the steers remained together throughout the experiment. Central pivot irrigation was used, and rotational grazing management was implemented with a fixed stocking rate. The pasture was established in the year 2008 after correcting for soil pH. It consisted of a diverse mix of species, including Orchard grass (*Dactylis glomerata*), Meadow brome (*Bromus commutatus*), Tall fescue (*Festuca arundinacea*, endophyte free), Sainfoin (Onobrychis), Birds foot trefoil (*Lotus corniculatus*), and Red clover (*Trifolium pratense*). Before the beginning of the grazing season, the area was cultivated with a drag arrow, followed by fertilization. Nitrogen and potassium were applied at a rate of 56 and 19 kg/ha, respectively.

To evaluate forage mass availability and quality in each pasture strip, samples were systematically collected from four random locations arranged in a pattern resembling the letter “N.” To achieve this, a quadrat metal frame measuring 0.5 × 0.5 m was positioned at each sampling point. The height of three plants inside the frame was measured from ground level to the curvature of the most recently expanded leaf, using a ruler (1 m in length). Subsequently, the grass was clipped to a height of 5 cm above the ground, and the harvested material was weighed. Finally, the four samples were combined to create a single mixed sample. From each mixed sample, two subsamples were extracted for specific analyses. One subsample was used to determine the dry matter content by subjecting it to drying in an oven at 65 °C for 72 h. Another subsample was reserved for subsequent chemical composition analyses. In addition to the routine sampling described above, control pellets and pellets mix samples were collected on a weekly basis. All forage samples were submitted for analysis to Cumberland Valley Analytical Services (Waynesboro, PA, USA). The comprehensive analysis covered various parameters, including dry matter, crude protein, soluble protein, rumen degradable protein, acid detergent fiber, neutral detergent fiber, nonfiber carbohydrate, ash, fat, lignin, total digestible nutrients, and mineral content (calcium, phosphorus, magnesium, potassium, sodium, iron, manganese, and copper). Detailed information about the laboratory procedures can be accessed via the following link: https://www.foragelab.com/Resources/Lab-Procedures (accessed on 30 October 2023).

### Statistical Analyses.

Sample size calculations and statistical analysis were conducted using SAS 9.4 (SAS Inst, Inc., Cary, NC, USA). Prior to analysis, the normality of the residuals was assessed using the Shapiro–Wilk test (PROC UNIVARIATE). The data were then subjected to analysis using a mixed models approach (PROC MIXED). To determine the appropriate covariance structure for each variable, we evaluated 15 different covariance structures. The selection of the best-fitting structure for each variable was based on the lowest value of the corrected Akaike information criteria (44).

Inspection of LW data was used to identify animals with atypical weight changes, which were subsequently excluded from the database. After excluding these animals, power analysis was conducted to ensure sufficient statistical power for detecting meaningful differences. An ANOVA followed by Tukey’s HSD test was performed to identify significant differences in bromoform intake over time for the treatment group.

The data were analyzed using a mixed model approach. This involved constructing models with fixed effects for treatment, time (experimental week), covariates, and the interaction between treatment and time. Random effects were incorporated for individual animals nested within phases to account for repeated measures. The phases (ramp-up, optimal, and decreasing) were incorporated to capture the temporal variability in bromoform intake and its impact on CH_4_ emissions and other variables. Each variable of interest was analyzed individually, including ILW, FLW, ADG, pDMI, CH_4_ emissions (CH_4_ in g/d, g/kg liveweight, g/kg ADG, and g/kg pDMI), CO_2_ emissions (CO_2_ in g/d, g/kg pDMI), and H_2_ emissions (H_2_ in g/d, g/kg pDMI). To account for baseline differences between animals, covariates for all variables (ILW, FLW, ADG, pDMI, etc.) were calculated using data from the initial time periods (Weeks 1 and 2). An autoregressive heterogeneous covariance structure [ARH (1)] was used to model the correlation between repeated measures over time. Least squares means were calculated for each treatment and time point, and the interaction effects were examined using the “SLICE” option to assess simple effects at each time point. Pairwise comparisons of means were performed with confidence limits to determine statistically significant differences. Significant differences among treatments were declared at *P* ≤ 0.05.

## Data Availability

All study data are included in the main text.
